# Specific prodromal symptoms in patients with acute coronary syndrome

**DOI:** 10.1002/nop2.663

**Published:** 2020-10-19

**Authors:** Mehdi Heidarzadeh, Shahla Elyaszadeh, Behrouz Dadkhah, Hossein Doustkami

**Affiliations:** ^1^ Department of Medical‐Surgical Nursing Nursing & Midwifery School Ardabil University of Medical Sciences Ardabil Iran; ^2^ Ardabil University of Medical Sciences Ardabil Iran; ^3^ Department of Cardiology School of Medicine Ardabil University of Medical Sciences Ardabil Iran

**Keywords:** acute coronary syndrome, ischemic heart diseases, nurses, nursing, prodromal symptoms

## Abstract

**Aims:**

Assessing the prodromal cardiac symptoms in patients with acute coronary syndrome (ACS) and compare them with healthy population.

**Background:**

Identifying specific prodromal cardiac symptoms can play an important role in screening people at risk.

**Design:**

A comparative study of prodromal symptoms.

**Methods:**

In this comparison study, an 80‐item checklist of prodromal symptoms was designed and completed by 337 participants in three groups (Patient group I, Patient group II and Healthy group). All participants were studied over a period of six months (from May to October 2017). SPSS‐15 software was used to analyse the data.

**Results:**

The symptoms of pain/discomfort in chest, pain/discomfort centred in the superior part of chest, pain/discomfort in the left breast and numbness or burning of both arms were the most important symptoms to predict ACS incidence in the two patient groups (odds ratio > 4 and *p* ≤ .05).

## 1 INTRODUCTION

Cardiovascular diseases are the world's leading cause of death (World Health Organization. [WHO], 2017), with estimated annual mortality rate of 17.3 million people, which is expected to exceed 23.6 million people by 2030 (Mozaffarian et al., 2015). Acute coronary syndrome (ACS) as a subset of cardiovascular diseases often is accompanied by clinical symptoms such as chest pain or discomfort, shortness of breath and fatigue (Kasper et al., 2015). Nurses can play an important role in reducing mortality and irreparable complications by early diagnosis of ACS and timely therapeutic and diagnostic measures (Canto et al., 2014; McSweeney et al., 2014, 2017). Getting know about “prodromal symptoms” can help nurses timely identify people at risk with minimum damage. In fact, prodromal symptoms are warning signs of an impending ACS (McSweeney et al., 2017) and signs of an impendent cardiac event that have a new onset or change in severity or frequency before the cardiac event and then disappear or return to its previous levels (McSweeney et al., 2003). Forty‐nine to ninety‐five per cent of the people at risk experience some undiagnosed symptoms as prodromal symptoms days, weeks, months and even 2 years before the onset of cardiac events (Lee et al., 2020; O'Keefe‐Mccarthy et al., 2019; Soltani et al., 2016).

## 2 BACKGROUND

O'Keefe‐McCarthy and Ready (2016) investigated the prevalence of heart disease prodromal symptoms in a review study and reported anxiety, unusual fatigue, pain or discomfort in arms etc. However, McSweeney et al. (2017) showed that the most common prodromal symptoms are not necessarily the most predictive symptoms of cardiac events. Despite the importance of the study by McSweeney et al. (2017), they examined the predictive properties of prodromal symptoms of cardiac events only among females and there is no information available on the specific prodromal symptoms among males. In general, not many studies have examined the specific prodromal symptoms in both genders.

Also, many factors can affect the predictive power of each of the prodromal symptoms. For example, previous studies have shown prodromal symptom in patients with ACS, but these symptoms may be related to other factors such as demographic characteristics and underlying diseases (Elyaszadeh et al., 2018). Therefore, it is important to evaluate these symptoms in other population especially in people without ACS experience.

The present study has two research questions as follows: (a) What are the prevalence of prodromal symptoms in patients (men and women) with ACS? (b) In compared with people without ACS (healthy population), which of these prodromal symptoms are specific for patients with ACS?

## 3 METHODS

### 3.1 Design

This comparison study was conducted to assess the prodromal cardiac symptoms in ACS patients in Imam Khomeini Hospital, Ardabil, Iran.

### 3.2 Participants

The 337 participants were divided into three groups: two patient groups diagnosed with ACS by a cardiologist at CCU and cardiac wards including 111 patients without a history of IHD as Patient group I and 107 with a history of myocardial infarction or angina pectoris as Patient group II. The third group included 119 patients with no ACS presenting to the heart clinics for heart checkup and were diagnosed as healthy after cardiac assessment by a cardiologist (healthy group). The inclusion criteria for Patient groups I and II were as follows: (a) definitive diagnosis of ACS by a cardiologist; (b) ability to speak Persian language; (c) normal cognitive status (according to medical history and clinical records); (d) favourable and stable physical conditions for conducting interviews; and (e) no history of IHD in Patient group I and a history of IHD in patient Group II. The inclusion criteria for the Healthy group were as follows: (a) healthy in terms of cardiac status (examined by a cardiologist based on physical examinations and diagnostic tests (echocardiogram, exercise treadmill test and electrocardiography) and no ACS incidence for at least 3 months after completing the questionnaire by researchers' follow‐up); (b) ability to speak Persian language; (c) possibility to follow‐up for the onset of ACS up to 3 months after sampling; and (d) normal cognitive status (according to medical history and clinical records).

### 3.3 Instrument and data collection

Data were collected with a demographic questionnaire and prodromal symptoms questionnaire. The prodromal symptoms questionnaire was designed in two phases. In the first step, a review of printed and electronic literature yielded 73 prodromal symptoms among ACS patients (Bahr et al., 2000; Bonow et al., 2011; Cole et al., 2012; Graham et al., 2008; Hofgren et al., 1995; Løvlien et al., 2009; McSweeney et al., 2003; McSweeney & Crane, 2000; Milner et al., 2001; Norris et al., 2008; O'Keefe‐McCarthy & Guo, 2016; Ottolini et al., 2005).

In the second step, for developing the questionnaire, a qualitative assessment was performed.

In the qualitative phase, 30 patients with ACS (15 men & 15 women) after receiving enough information about the study goal filled the 73‐item questionnaire prepared in the first step. Then, they were asked to express their experience of other symptoms before the incidence of ACS which they believed were related to their heart attack but were not listed in the 73‐item list. During analyses of their statements, seven new prodromal symptoms were extracted including tingling toes, hot flashes, a feeling of heat on the foot, dry mouth, foot pain, numbness toes and knee pain. At the end of this part, an 80‐item questionnaire was developed.

In addition to examining the incidence of 80 symptoms, this questionnaire assessed their severity and frequency. For this purpose, the mean score of each symptom was scored based on the incidence, severity and frequency and ranged from 0–10 for each item based on incidence (0 and 1 for No and Yes, respectively), intensity (1, 2 and 3 for mild, moderate and severe, respectively) and frequency (6, 5, 4, 3, 2 and 1 for daily, several times a week, once a week, 2–3 times a month, once a month and less than once a month, respectively). High score of each item shows more severity and frequency of the symptom. This way of scoring has been introduced by O'Keefe‐McCarthy and Guo (2016). For both patient Groups I and II, items were asked retrospectively with a focus on the past 3 months. Meanwhile, the subjects in the healthy group were monitored prospectively for 3 months after sampling in terms of ACS incidence and after the three‐month follow‐up, none of the participants in the healthy group had ACS.

The content validity index (CVI) and content validity ratio (CVR) were used to determine the content validity of the questionnaire. The questionnaire was distributed to five nursing faculty members and five expert clinicians in the cardiovascular field. The CVI and CVR scores for the total scale were determined as 0.8 and 0.79, respectively. To determine the internal consistency reliability, the Cronbach's alpha coefficient was calculated 0.8 among 30 patients with ACS presenting.

All the participants were interviewed to complete the questionnaires in bedside (at CCU and cardiac wards) or inside the nursing room (at heart clinic). Every interview and completing the questionnaires lasted approximately 20–30 min in all groups.

### 3.4 Statistical analyses

Data were analysed using descriptive and inferential statistics. Analysis of variance (ANOVA) with post hoc Tukey test was used for determining any significant differences between the scores of every symptom in the groups. Also, logistic regression was used to calculate odds ratios at a 95% confidence interval for the association between the presence/absence of each of the prodromal symptoms with ACS incidence (and lack of it in healthy population) as a dependent variable and each symptom tested separately. All the symptoms were analysed twice; first, we considered group I against the healthy group as a dependent variable; then, we did it in group II and healthy group in the same way. Data were analysed in SPSS‐15 software.

### 3.5 Ethical considerations

This study was approved by the Ethics Committee of the Ardabil University of Medical Sciences. One of the researchers (first author) was referred to the clinics and invited eligible participants to the study (May–October in 2017). Then, willing participants were also sked to answer questionnaire by interview. Before completing the questionnaires, participants were briefed on the goals and method of the study before signing a written consent. Patients were assured that the study would not disturb their treatment process, the collected information would be completely confidential and the results would be published collectively.

## 4 RESULTS

### 4.1 Sample description

A total of 337 subjects were recruited in three groups of Healthy (*N* = 119), Patient I (*N* = 111) and Patient II (*N* = 107) for data analysis. Healthy group did not differ significantly with any of the Patient groups I and II in terms of age, sex and underlying diseases (*p* > .05). Demographic characteristics and medical history of the groups are presented in Table 1.

**TABLE 1:** Demographic characteristics and medical history of the participants by groups

Parameter	Healthy group *n* (%)	Patient group I *n* (%)	Patient group II *n* (%)	*p*‐value
Mean Age, year (*SD*)	63.15 (11.34)	62.14 (10.72)	64.75 (10.45)	.206^a^
Gender				
Female	58 (48.7)	50 (45)	51 (47.7)	.848^b^
Male	61 (51.3)	61 (55)	56 (52.3)	
ACS				
Myocardial infarction	—	59 (53.2)	45 (42.1)	—
Unstable angina	—	52 (46.8)	62 (57.9)	
Medical history				
Hypertension	83 (69.7)	73 (65.8)	84 (78.5)	.105^b^
Diabetes	31 (26.1)	29 (26.1)	33 (30.8)	.662^b^
Hyperlipidemia	40 (33.6)	37 (33.3)	41 (38.3)	.686^b^
Low back pain	18 (15.1)	11 (9.9)	14 (13.1)	.492^b^
Rheumatoid arthritis	27 (22.7)	20 (18)	18 (16.8)	.492^b^
Thyroid condition	9 (7.6)	7 (6.3)	6 (5.6)	.833^b^
IBS, Peptic Ulcer, Oesophageal Reflux	4 (3.4)	3 (2.7)	3 (2.8)	.951^b^
COPD, Asthma	3 (2.5)	3 (2.7)	6 (5.6)	.408^b^
Sinusitis, migraine	6 (5.6)	5 (4.5)	3 (2.5)	.480^b^
BPH	4 (6.6)	3 (4.9)	6 (10.7)	.478^b^
Other	12 (10.1)	11 (9.9)	13 (12.1)	.837^b^

^a^ Using ANOVA.

^b^ Using chi‐square tests; other: depression, anaemia, cerebrovascular accident, epilepsy, cholecystitis, fatty liver disease and a cold. Patient group I: participants with ACS incidence and without a history of ischaemic heart disease, patient group II: participants with ACS incidence and with a history of ischaemic heart disease; healthy group: participants without ACS incidence; ACS, acute coronary syndrome; IBS, irritable bowel syndrome; COPD, chronic obstructive pulmonary disease; BPH, benign prostatic hyperplasia; *SD*, standard deviation.

In this study, 80 prodromal symptoms were analysed in terms of frequency and mean score in three groups. By comparing the results (frequency and mean score of prodromal symptoms) in patient groups with healthy group, the true value of each symptom became clear.

### 4.2 Comparing the frequency

Logistic regression was used for investigating the predictive power of the prodromal symptoms. To this end, the frequency of each symptom in the Patient group I and Patient group II was compared with its frequency in the healthy group. Based on the results, the frequency of 24 symptoms in the Patient group I was significantly higher than those of the Healthy group. Furthermore, the Patient group II had higher scores in the same 24 symptoms plus 15 other symptoms (a total of 39 symptoms) compared with the Healthy group (Table 2).

**TABLE 2:** Prevalence and mean score of each prodromal symptom by groups and significant association of the prodromal symptom with incidence of ACS

Symptom	Frequency *n* (%)			Score Mean (*SD*)		
	Patient group I (*N* = 111)	Patient group II (*N* = 107)	Healthy group (*N* = 119)	Patient group I (*N* = 111)	Patient group II (*N* = 107)	Healthy group (*N* = 119)
Pain/discomfort in chest	66 (59.5)***	88 (82.2) ***	17 (14.3)	4.55 (4.02)^a^	6.78 (3.58)^a^	0.82 (2.19)
Pain/discomfort centred in the superior part of chest	51 (45.9)***	66 (61.7)***	16 (13.4)	3.52 (4.01)^a^	5.06 (4.23)^a^	0.80 (2.18)
Pain/discomfort in left breast	32 (28.8)***	40 (37.4)***	9 (7.6)	2.29 (3.75)^a^	3.01 (4.05)^a^	0.44 (1.60)
Pain/discomfort centred in left part of chest	19 (17.1)***	35 (32.7)***	3 (2.5)	1.28 (2.93)^a^	2.60 (3.89)^a^	0.14 (1.02)
Vomiting	22 (19.8)***	16 (15)**	7 (5.9)	1.43 (3.01)^a^	0.77 (2.00)	0.33 (1.37)
Numbness or burning of both arms	16 (14.4)***	21 (19.6)***	4 (3.4)	1.08 (2.77)^a^	1.65 (3.44)^a^	0.25 (1.42)
Pain/discomfort in back, between/ under shoulder blades	68 (61.3)**	70 (65.4)***	34 (28.6)	4.98 (4.18)^a^	5.46 (4.20)^a^	1.97 (3.24)
Pain/discomfort in both arms	43 (38.7)**	44 (41.1)**	21 (17.6)	3.29 (4.30)^a^	3.53 (4.39)^a^	1.36 (3.05)
Pain/discomfort in neck/throat	54 (48.6)**	58 (54.2)**	37 (31.1)	3.85 (4.16)^a^	4.23 (4.17)^a^	2.31 (3.64)
Diaphoresis	69 (62.2)**	75 (70.1)**	53 (44.5)	5.18 (4.28)^a^	6.05 (4.22)^a^	3.76 (4.40)
Shortness of breath	52 (46.8)**	65 (60.7)***	34 (28.6)	3.78 (4.23)^a^	5.18 (4.38)^a^	2.05 (3.37)
Arms ache	33 (29.7)**	40 (37.4)***	15 (12.6)	2.60(4.07)^a^	3.42 (4.51)^a^	1.02 (2.76)
Difficulty breathing at night	30 (27)**	38 (35.5)***	13 (10.9)	2.19 (3.76)^a^	3.04 (4.27)^a^	0.70 (2.06)
Nausea	28 (25.2)**	25 (23.4)**	14 (11.8)	1.91 (3.43)^a^	1.63 (3.10)	0.85 (2.44)
Orthopnea	28 (25.2)**	40 (37.4) ***	15 (12.6)	2.05 (3.63)^a^	3.24 (4.31)^a^	0.76 (2.04)
Pain/discomfort in jaw/teeth	21 (18.9)**	31 (29)***	11 (9.2)	1.49 (3.20)	2.28 (3.70)^a^	0.63 (2.08)
Panic	10 (9)**	11 (10.3)***	3 (2.5)	0.55 (1.87)	0.77 (2.35)^a^	0.16 (1.10)
Headache frequency change(Increased frequency of headaches)	28 (25.2)**	33 (30.8)**	17 (14.3)	2.14 (3.80)	2.71 (4.13)^a^	1.04 (2.65)
Headache intensity change(Increased intensity of headaches)	28 (25.2)**	22 (20.6)**	13 (10.9)	2.08 (3.69)^a^	1.92 (3.80)^a^	0.86 (2.51)
Sleep disturbance	62 (55.9)*	73 (68.2)**	50 (42)	4.75 (4.42)^a^	5.95 (4.28)^a^	3.19 (3.94)
Arms weak/heavy	49 (44.1)**	53 (49.5)**	28 (23.5)	3.82 (4.38)^a^	4.36 (4.53)^a^	1.86 (3.49)
Pain/discomfort at top of shoulders	46 (41.4)*	58 (54.2)**	32 (26.9)	3.46 (4.28)^a^	4.49 (4.35)^a^	2.03 (3.48)
Frequent indigestion	42 (37.8)*	47 (43.9)**	29 (24.4)	3.04 (4.10)	3.77 (4.43)^a^	1.87 (3.41)
A feeling of fatigue at waking up	43 (38.7)*	40 (37.4)*	30 (25.2)	3.30 (4.27)^a^	3.28 (4.32)^a^	1.98 (3.50)
Unusual fatigue	60 (54.1)	69 (64.5)**	55 (46.2)	4.56(4.41)	5.69 (4.38)^a^	3.71 (4.17)
Heart racing	51 (45.9)	68 (63.9)**	43 (36.1)	3.65 (4.10)^a^	5.20 (4.20)^a^	2.39 (3.42)
Waking up repeatedly during the night	46 (41.4)	63 (58.9)**	46 (38.7)	3.51 (4.32)	5.27 (4.55)^a^	3.09 (4.03)
Dizziness	45 (40.5)	58 (54.2)**	43 (36.1)	2.85 (3.71)	4.30 (4.15)^a^	2.49 (3.47)
Insomnia	47 (42.3)	55 (51.4)**	39 (32.8)	3.67 (4.43)	4.66 (4.67)^a^	2.50 (3.78)
Trouble falling asleep	45 (40.5)	56 (52.3)**	37 (31.1)	3.57 (4.43)	4.74 (4.65)^a^	2.50 (3.87)
Loss of appetite	32 (28.8)	45 (42.1)**	23 (19.3)	2.49 (4.03)	3.82 (4.60)^a^	1.59 (3.55)
Numbness or burning of fingers on both hands	31 (27.9)	41 (38.3)**	21 (17.6)	2.22 (3.70)	3.07 (4.09)^a^	1.34 (2.97)
Hands/arms tingling	28 (25.2)	42 (39.3)**	27 (22.7)	2.09 (3.61)	3.13 (7.05)^a^	1.70 (3.29)
Confusion	8 (7.2)	15 (14)**	6 (5)	0.42 (1.62)	1.05 (2.70)^a^	0.26 (1.17)
Agoraphobia	8 (7.2)	10 (9.3)**	2 (1.7)	0.48 (1.81)	0.78 (2.51)^a^	0.15 (1.17)
Headaches	48 (43.2)	61 (57)*	50 (42)	3.24 (3.97)	4.35 (4.05)^a^	2.79 (3.54)
Heartburn	48 (43.2)	48 (44.9)*	38 (31.9)	3.40 (4.16)	3.60 (4.19)	2.26 (3.45)
Sputum	43 (38.7)	48 (44.9)*	36 (30.3)	3.08 (4.07)	3.83 (4.37)^a^	2.13 (3.47)
Cough	35 (31.5)	41 (38.3)*	31 (26.1)	2.47 (3.77)	3.07 (4.13)^a^	1.75 (3.18)
Unusually located aches and/or pains	69 (62.2)	63 (62.2)	64 (53.8)	5 (4.12)	5.13 (4.45)	4.4 (4.35)
Nervousness	61 (55)	57 (53.3)	71 (59.7)	4.49 (4.27)	4.34 (4.27)	4.31 (3.89)
Anxiety	58 (52.3)	62 (57.9)	66 (55.5)	4.26 (4.29)	4.72 (4.26)	3.96 (3.87)
Stress	49 (44.1)	41 (38.3)	54 (45.4)	3.78 (4.40)	3.34 (4.37)	3.44 (4.07)
Changes in thinking or remembering	48 (43.2)	45 (42.1)	56 (47.1)	3.33 (4.07)	3.55 (4.30)	3.38 (3.81)
Irritation	47 (42.3)	40 (37.4)	36 (30.3)	3.48 (4.24)	3.24 (4.33)	2.38 (3.78)
Pain in back (backache)	47 (42.3)	43 (40.2)	59 (49.6)	3.28 (4.10)	3.50 (4.41)	3.86 (4.20)
Gastric reflux	45 (40.5)	44 (41.1)	46 (38.7)	3.08 (3.96)	2.99 (3.82)^a^	2.59 (3.53)
Apprehension	44 (39.6)	36 (33.6)	47 (39.5)	3.09 (4.01)	2.88 (4.16)	2.93 (3.91)
Weakness	43 (38.7)	50 (46.7)	42 (35.3)	3.09 (4.05)	3.91 (4.38)^a^	2.55 (3.63)
General malaise	40 (36)	34 (31.8)	41 (34.5)	2.80 (3.89)	2.81 (4.21)	2.56 (3.77)
Pain/ discomfort in legs	40 (36)	35 (32.7)	60 (50.4)	3.13 (4.32)	2.92 (4.29)^a^	4.29 (4.44)
Hot flashes	40 (36)	41 (38.3)	40 (33.6)	2.97 (4.14)	3.25 (4.29)	2.78 (4.07)
Flatulence	35 (31.5)	42 (39.3)	46 (38.7)	2.70 (4.08)	3.40 (4.35)	2.85 (3.79)
Vision change	34 (30.6)	44 (41.1)	39 (32.8)	2.39 (3.72)	3.03 (3.86)	2.26 (3.44)
Demoralization	28 (25.2)	36 (33.6)	36 (30.3)	2.08 (3.71)	2.83 (4.13)	2.24 (3.58)
A feeling of hopelessness	27 (24.3)	20 (18.7)	23 (19.3)	1.99 (3.63)	1.56 (3.41)	1.52 (3.25)
Depression	26 (23.4)	30 (28)	31 (26.1)	1.94 (3.57)	2.29 (3.81)	1.91 (3.37)
Pain/ discomfort in left arm or shoulder	22 (19.8)	18 (16.8)	13 (10.9)	1.52 (3.14)	1.44 (3.28)	0.84 (2.43)
Knee (s) pain	19 (17.1)	10 (9.3)	17 (14.3)	2.97 (4.14)	0.88 (2.76)	1.23 (3.10)
Wanting to be dead at times	18 (16.2)	17 (15.9)	12 (10.1)	1.20 (2.93)	1.30 (3.12)	0.77 (2.44)
Hand (s)/arm (s) oedema	17 (15.3)	18 (16.8)	12 (10.1)	1.05 (2.72)	1.34 (3.13)	0.79 (2.47)
Dry mouth	17 (15.3)	11 (10.3)	16 (13.4)	1.29 (3.09)	0.81 (2.50)	1.08 (2.80)
Not being able to cope with everyday problems as well as before	17 (15.3)	22 (20.6)	16 (13.4)	1.23 (3.01)	1.73 (3.49)	1.01 (2.66)
Numbness of toes	16 (14.4)	18 (16.8)	21 (17.6)	1.18 (2.97)	1.52 (3.45)	1.68 (3.24)
Tingling toes	16 (14.4)	18 (16.8)	27 (22.7)	1.48(3.30)	1.28 (3.15)	1.68 (3.24)
A feeling of heat on the foot	14 (12.6)	15 (14)	32 (26.9)	1.06 (2.89)^a^	1.23 (3.15)	2.23 (3.80)
Numbness or burning of fingers left hand	13 (11.7)	13 (12.1)	7 (5.9)	0.87 (2.57)	0.91 (2.63)^a^	0.15 (1.05)
Pain/ discomfort in the general chest	12 (10.8)	17 (15.9)	0 (0)	0.86 (2.58)^a^	1.36 (3.22)^a^	0 (0)
Increasing difficulty in concentrating on a single subject for long	9 (8.1)	11 (10.3)	11 (9.2)	0.65 (2.23)	0.81 (2.49)	0.63 (2.10)
Leg/foot oedema	8 (7.2)	10 (9.3)	17 (14.3)	0.50 (1.95)	0.74(2.42)	1.02 (2.64)
Numbness or burning of fingers right hand	7 (6.3)	10 (9.3)	8 (6.7)	0.45 (1.87)	0.66 (2.27)	0.30 (1.50)
Pain/ discomfort in right arm or shoulder	7 (6.3)	6 (5.6)	19 (16)	0.56 (2.19)	0.47 (1.94)^a^	1.31 (3.08)
Foot pain	7 (6.3)	4 (3.7)	5 (4.2)	0.58 (2.28)	0.30 (1.55)	0.38 (1.83)
Abdominal discomfort	6 (5.4)	12 (11.2)	15 (12.6)	0.44 (1.90)	0.87 (2.55)	0.87 (2.35)
Numbness or burning of left arm	6 (5.4)	7 (6.5)	2 (1.7)	0.39 (1.67)	0.50 (2.00)	0.05 (0.55)
Numbness or burning of right arm	4 (3.6)	2 (1.9)	2 (1.7)	0.31 (1.60)	0.18 (1.29)	0.13 (0.97)
Pain/ discomfort centred right part of the chest	4 (3.6)	2 (1.9)	1 (0.8)	0.25 (1.35)	0.16 (1.17)	0.05 (0.55)
Syncope	2 (1.8)	6 (5.6)	1 (0.8)	0.08 (0.63)	0.23 (1.00)	0.07(0.73)
Oedema in other part of the body(except leg, foot, hand, arm)	2 (1.8)	3 (2.8)	0 (0)	0.13 (1.06)	0.21 (1.30)	0 (0)
Pain/discomfort centred inferior part of the chest	1 (0.9)	4 (3.7)	0 (0)	0.08 (85)	0.30 (1.54)	0 (0)

Note

Patient group I: participants with ACS incidence and without a history of ischaemic heart disease; patient group II: participants with ACS incidence and with a history of ischaemic heart disease; healthy group: participants without ACS incidence; *SD*: standard deviation.

^a^ According to ANOVA with post hoc Tukey test, the marked values significantly are higher in the patients groups in comparison with healthy group.

* Significant association of the prodromal symptom with incidence of ACS with odd ratio of less than 2

** Significant association of the prodromal symptom with incidence of ACS with odd ratio of 2–4

*** Significant association of the prodromal symptom with incidence of ACS with odd ratio of more than 4.

### 4.3 Comparing the mean score

For more analyses, significant differences between the mean score of every prodromal symptom in three groups were calculated. ANOVA with post hoc Tukey test showed that the scores of 23 prodromal symptoms (20 of 24 predictive symptoms) in patients group I and 42 (36 of 39 predictive symptoms) in patients group II were significantly higher than healthy group (Table 2).

## 5 DISCUSSION

The results of the study showed that 24 specific symptoms predicting ACS both in patients with IHD and patients without IHD, some of which are pain/discomfort in chest, heart racing, diaphoresis, vomiting and shortness of breath. Some other researchers found contradictory results in their study. For example, they have reported vomiting, heart racing and diaphoresis as the non‐specific prodromal symptoms (McSweeney et al., 2014; O'Keefe‐McCarthy & Guo, 2016). The difference in the characteristics of the participants and data analysis might attribute to this inconsistency. In the most of these studies, only female enrolled and higher probability of occurrence and high frequency of symptoms were the most criteria for determining specific symptoms in these studies. In the present study, both men and women enrolled. In addition, indicators such their higher probability of occurrence compared with the healthy subjects their abundance and mean score of symptoms (severity and frequency) were also calculated.

The results of this study showed that a history of ischaemic heart disease can be effective in the type of symptom experienced and its predictive power. For example, among common predictive symptoms in patient groups, vomiting in people without a history of IHD (patient group I) was higher than in those with a history of IHD (patient group II) and, on the other hand, shortness of breath, discomfort in the arms, difficulty breathing at night, orthopnea, pain/discomfort in jaw/teeth and panic in people with a history of IHD (patient group II) had a higher predictive power than in those without a history of IHD (patient group I). Also, individuals with a history of IHD (patient group II) experienced 15 other symptoms in addition to the 24 common symptoms (Table 2).

Few studies have been conducted on the specific prodromal symptoms with a focus on the history of heart disease. In one of the few studies in this area, McSweeney et al. (2017) found similar results in a study to identify predictive prodromal symptoms of cardiac events in women without a history of heart diseases. In their study, similar to the present study, the symptoms of discomfort in arms and discomfort in teeth/jaw were significantly related to the incidence of cardiac events and symptoms such as pain in feet, back pain, pain/discomfort in the general chest, vision change, changes in thinking or remembering, anxiety, abdominal discomfort and pain/discomfort centred in the right part of the chest were not related to ACS incidence (McSweeney et al., 2017). Despite these similarities, some results in the present study were contradictory to those of McSweeney's study in that symptoms such as discomfort in arms, difficult breathing at night, shortness of breath, numbness in both arms, weak/heavy feeling in arms, sleep disturbances, frequent dyspepsia, pain/discomfort at top of shoulders, pain/discomfort in neck/throat, headache intensity change, headache frequency change, pain/discomfort centred in the superior part of chest, loss of appetite, cough, dizziness and unusual fatigue were correlated with ACS incidence in the present study, while these symptoms were not significantly correlated with ACS in the study by McSweeney et al. (2017).

We found that the prevalence of a symptom does not indicate the specificity of the symptom, since symptoms such as unusually located aches and/or pains, nervousness, anxiety, changes in thinking or remembering and back pain, although highly prevalent in patient groups, were prevalent in the healthy group, too and their scores were close to those of the patient groups. Due to their nature, these symptoms appear to lack the credibility of ACS prediction. For instance, symptoms of anxiety and unusually located aches and/or pains have several causes, including physiological, situational and psychological factors, in addition to heart status (Elyaszadeh et al., 2018; Faravelli et al., 2013; Kasper et al., 2015). Impending ACS is just one of the factors affecting their experience. Therefore, people who are not at risk of ACS can also experience them due to a variety of other causes and experiencing these symptoms is not specifically related to an impending ACS.

### 5.1 Limitations

The limitations of the study included the use of convenience sampling, which makes the generalization of the results subject to caution. The gold standard for ruling out coronary artery disease is angiography, but it was not possible to perform it in the healthy group to rule out the coronary artery disease. As a result, according to a cardiologist, we only used diagnostic tests (echocardiogram, exercise treadmill test and electrocardiography) and physical examination; also, subjects were monitored for ACS up to 3 months after sampling. Retrospective design is another limitation which led participants recall recurrent symptoms in previous months and participants experiencing ACS remember symptoms better than who did not. Although we tried to set a time limit for remembering experienced symptoms up to three months ago which can decrease the chance of forgetfulness. On the other hand, researches support the accurate recall of significant medical life events up to 6 years (Githens et al., 1993), it is possible that some details of the symptoms were forgotten.

Another limitation of this study was conducting the research in the community of ACS patients in Ardabil, Iran, which limits the generalizability of the results to other populations, and it is suggested that the specificity of these symptoms be investigated in other populations.

## 6 CONCLUSION

The present study showed that both patients group I and II had experienced same prodromal symptom, but the mean score of them (severity and frequency) was different. Also, individuals with a history of IHD (patient group II) reported experiencing of 15 more specific symptoms than patient group I.

According to this study, although there are some symptoms experienced in both patients group, the healthy population experienced them, too. It indicates, these symptoms maybe emerge by other causes than ischaemic heart diseases.

## 7 IMPLICATIONS FOR PRACTICE

The findings of this study have certain applications in nursing practice and research. We tried to assess all possible prodromal symptoms collected by literature review and qualitative interviews and to give evidence for frequency and mean score of all 80 prodromal symptoms. Comparing both frequency and mean score of prodromal symptoms in patient groups with healthy group, provides valuable information about predicting power of prodromal symptoms and determined the specific prodromal symptoms in each group.

This study also showed that the frequency and mean score of prodromal symptoms cannot be appropriate criteria for predicting ACS, so it is necessary to consider the predicting value of the symptoms. Knowing about these specific prodromal symptoms by nurses, other healthcare providers and patients at risk of ACS can be helpful in the early diagnosis of IHD and prevent ACS, so this can lead to timely therapeutic measures and a reduction in irreparable complications and mortality from IHD.

Another application of the findings is that frequency and severity of each specific prodromal symptom in this study can provide well information for researchers to design or develop valid tools for predicting ACS in the people at risk of ACS.

## CONFLICTS OF INTEREST

The authors have no funding or conflicts of interest to disclose.

## Data Availability

The data used to support the findings of this study are available from the corresponding author on reasonable request.
